# Crystal structure and Hirshfeld surface studies of 4-bromo-2-chloro­phenyl (2*E*)-3-[4-(pent­yloxy)phen­yl]prop-2-enoate

**DOI:** 10.1107/S2056989025003317

**Published:** 2025-09-05

**Authors:** M. Harish Kumar, S. Santhosh Kumar, H. C. Devarajegowda, H. T. Srinivasa, B. S. Palakshamurthy

**Affiliations:** a Department of Physics Yuvaraja's College University of Mysore,Mysore 570005 Karnataka India; b Department of PG Studies and Research in Physics Albert Einstein Block UCS Tumkur University, Tumkur Karnataka-572103 India; c Raman Research Institute, C V Raman Avenue Sadashivanagar Bangalore Karnataka India; University of Buenos Aires, Argentina

**Keywords:** crystal structure, 4-bromo-2-chloro­phen­yl, Hirshfeld surface

## Abstract

The asymmetric unit of the compound C_20_H_20_BrClO_3_ contains one independent mol­ecule in which the aromatic rings are oriented at a dihedral angle of 83.30 (2)°. An intra­molecular C—H⋯O contact generates a five-membered *S*(5) ring motif. In the crystal, C—H⋯O hydrogen bonds link the mol­ecules through *R*^1^_2_(6), *R*^2^_2_(10), *R*^2^_2_(14) hydrogen-bond motifs. The structure is consolidated by C—H⋯π inter­actions. A Hirshfeld surface analysis of the crystal structure indicates that the most important contributions for the crystal packing are from H⋯H (32.8%), C⋯H/H⋯C (28.1%), O⋯H/H⋯O (14.0%) Br⋯H/H⋯Br (12.5%) and Cl⋯H/H⋯Cl (10.6%) inter­actions.

## Abstract 2

In the title compound, the aromatic rings are oriented at a dihedral angle of 83.30 (2)°. An intra­molecular C—H⋯O contact generates a five-membered *S*(5) ring motif. In the crystal, C—H⋯O hydrogen bonds link the mol­ecules through *R*^1^_2_(6), *R*^2^_2_(10), *R*^2^_2_(14) hydrogen-bond motifs.

## 1. Chemical context

4-Bromo-2-chloro­phenyl derivatives possess an inter­esting *in vitro* inhibitory activity on plasmodium falciparum bacteria and serve as the starting mol­ecule for structure-based design of novel inhibitors for anti-plasmodial and transmission-blocking agents (Vallone *et al.*, 2018 ▸). These compounds are known to exhibit anti-malarial activity (Kos *et al.*, 2022 ▸). Compounds having halogen atoms at the *ortho* and *meta* positions with respect to the bromine have also been found to exhibit anti-inflammatory activity (Hošek *et al.*, 2019 ▸). This class of compounds are very important for the design of drugs for the treatment of diseases such as dengue and chikungunya and furthermore, the introduction of alkyl groups into these compounds will induce better penetration capacity at the cellular level. Compounds obtained by combining 4-bromo-2-chloro­phenyl and (alk­yloxy)phenyl-derived mol­ecules are well known for their anti­microbial activity (Radwan *et al.*, 2014 ▸) and anti­tumor properties (Jung *et al.*, 2019 ▸; Pieters *et al.*, 1999 ▸). The role of alkyl groups in the various drug mol­ecules is to speed up the penetration of compounds into the cell *i.e.* into mitochondria. In this context, it is found that decyl­caffeic acid inhibits the growth of colorectal cancer cells (Lukáč *et al.*, 2024 ▸) and alkyl groups in cinnamic acid-based mol­ecules encourage anti­tuberculosis activity (De *et al.*, 2011 ▸). The alkyl group, which makes an amido links with various aromatic or heterocyclic rigid cores, will enhance the degree of inhibition activity of anti-inflammatory drugs (Matta *et al.*, 2020 ▸). Keeping these properties in mind, we decided to synthesize and study the title compound, which has both a rigid core (4-bromo-2-chloro­phen­yl) and an alk­yloxy chain linked through the ester group and present the results herein.
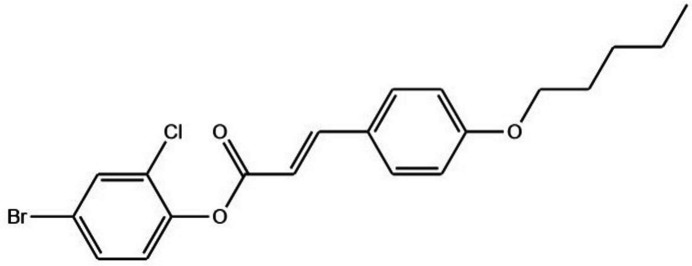


## 2. Structural commentary

**Figure 1:**
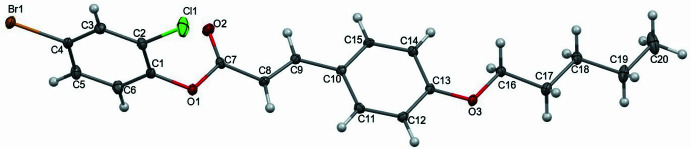
The mol­ecular structure of the title compound, showing displacement ellipsoids drawn at the 50% probability level.

**Figure 2:**
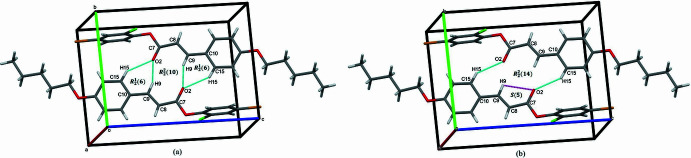
The mol­ecular packing of the title compound. Dashed lines indicate the C—H⋯O hydrogen-bonding inter­actions. The 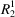
(6), 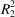
(10) (*a*) and 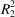
(14) (*b*) synthons are indicated by dotted pale-green lines and the *S*(5) ring is shown in pink (*b*).

The title compound (Fig. 1 ▸) crystallizes in space group *P*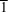. The dihedral angle between the 4-bromo-2-chloro­phenyl aromatic ring (C1–C6) and the aromatic ring of the pent­yloxy phenyl fragment (C10–C15) is 83.30 (2)°. The torsion angles C1—O1—C7—C8 and C13—O3—C16—C17 are 175.98 (14) and −179.32 (14)°, respectively, which are *anti-periplanar*. The H8—C8=C9—H9 atoms exhibit an *E*-configuration with a torsion angle of 178° and the mol­ecule is non planar with an r.m.s. deviation of 0.065 Å. An intra­molecular hydrogen-bond inter­action generates an *S*(5) motif (Fig. 2 ▸*b*).

## 3. Supra­molecular features

**Figure 3:**
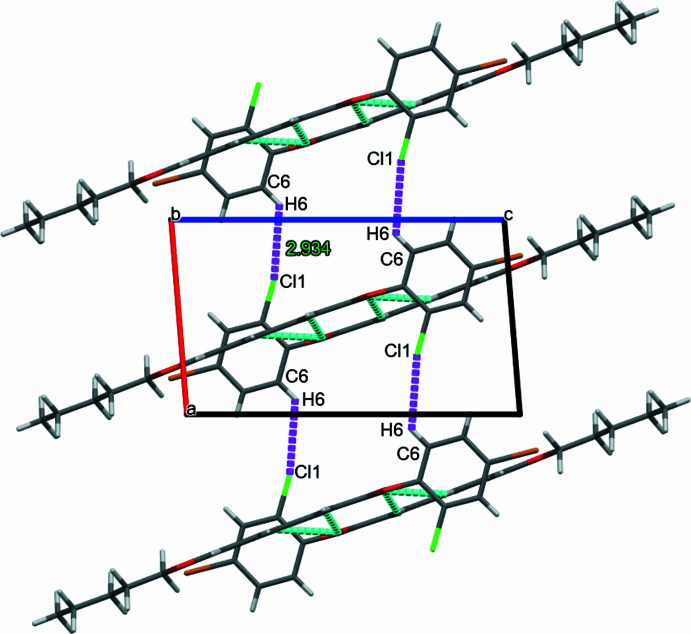
The mol­ecular packing of the title compound with weak C—H⋯Cl inter­actions indicated by magenta coloured dashed lines.

**Figure 4:**
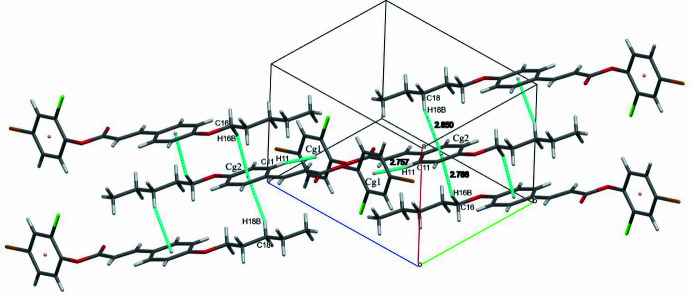
The mol­ecular packing of the title compound. Dashed lines indicate the C—H⋯π inter­actions.

In the crystal, C—H⋯O hydrogen bonds (Table 1 ▸, Fig. 2 ▸*a*) link the mol­ecules through 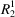
(6), 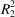
(10) and 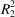
(14) cyclic hydrogen-bond motifs (Bernstein *et al.*, 1995 ▸), forming inversion dimers. Together these inter­actions generate mol­ecular sheets parallel to (010). The inversion dimers are linked through weak C—H⋯Cl inter­actions (Table 1 ▸) as shown in Fig. 3 ▸. The packing is further consolidated by C—H⋯π inter­actions (Fig. 4 ▸, Table 1 ▸).

## 4. Hirshfeld surface analysis

**Figure 5:**
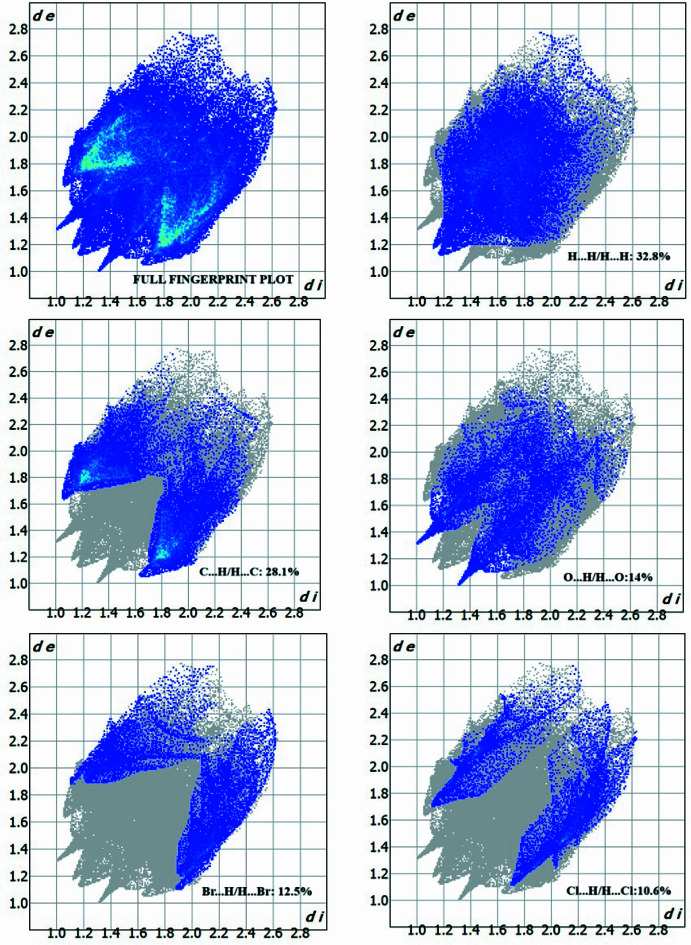
The two-dimensional fingerprint plots for the title compound, showing all inter­actions, and those delineated into H⋯H, C⋯H/H⋯C, O⋯H/H⋯O, Br⋯H/H⋯Br and Cl⋯H/H⋯Cl contacts.

Hirshfeld surface analysis (Hirshfeld, 1977 ▸; Spackman & Jayatilaka, 2009 ▸) was used to visualize and qu­antify inter­molecular inter­actions using *Crystal Explorer* (Spackman *et al.*, 2021 ▸). The two-dimensional fingerprint plots (Fig. 5 ▸) qu­anti­fying the various inter­molecular inter­actions indicate that the major contributions to the crystal packing of the title mol­ecule are from H⋯H (32.8%), C⋯ H/H ⋯C (28.1%), O⋯H/H⋯O (14.0%), Br⋯H/H⋯Br (12.5%) and Cl⋯H/H⋯Cl (10.6%) contacts.

## 5. Database survey

A search of the Cambridge Structural Database (CSD version 2.0.4, December 2019; Groom *et al.*, 2016 ▸) for mol­ecules containing the 4-bromo-2-chloro­phenyl moiety resulted in 15 matches. Of these, the six compounds with CSD codes EBEPUZ (Lehmler *et al.*, 2013 ▸), EJULUT (Dumitrescu *et al.*, 2020 ▸), ISOJUX (Reddy *et al.*, 2016 ▸), FANFOS (Sangeeta *et al.*, 2017 ▸) and VIDQUX (Mohan *et al.*, 2018 ▸) have either alk­yloxy or substituted aromatic or heterocyclic rings connected fragments that are found to be in the same plane. The dihedral angle made by these planes with the 4-bromo-2-chloro­phenyl moiety are 59.0, 75.3, 88.78, 37.47, and 2.99°, respectively, whereas in the title compound, the dihedral angle between the 4-bromo-2-chloro­phenyl and (pent­yloxy)phenyl­prop-2-enoate moieties is 82.15 (2)°. The torsion angle between the *ortho*-substituted chlorine atom and the first atom of the planar functional group in the above compounds is between 1 and 3° while in the title compound this torsion angle is 1.2 (2)°.

## 6. Synthesis and crystallization

A mixture of 4-bromo-2-chloro­phenol (0.208 g, 0.001 mol) and (*E*)-3-[4-(pent­yloxy)phen­yl]acrylic acid (0.234 g, 0.001 mol) was suspended in anhydrous chloro­form (10 ml). To this was added *N*,*N*-di­cyclo­hexyl­carbodi­imide (0.206 g, 0.001 mol) and 4-*N*,*N*-di­methyl­amino pyridine (5 mg) and the mixture stirred overnight at room temperature. The *N*,*N*-di­cyclo­hexyl urea formed was filtered off and the filtrate diluted with chloro­form (25 ml). This solution was washed successively with 5% aqueous acetic acid solution (2 × 25 ml) and water (2 × 25 ml) and dried on sodium sulfate*.* The residue obtained on removal of solvent was chromatographed on silica gel using chloro­form as eluent. Removal of solvent from the eluate afforded a white material that was crystallized from a ­chloro­form–petroleum ether mixture. Yield 75%. Elemental analysis calculated: C, 56.69; H, 4.76; Br, 18.86; Cl, 8.37; O, 11.33%; found: C, 56.71; H, 4.79; Cl, 8.42%, m.p. 371–373 K.

## 7. Refinement

Crystal data, data collection and structure refinement details are summarized in Table 2 ▸. H atoms were positioned geometrically (C—H = 0.93 Å) and refined as riding with *U*_iso_(H) = 1.2*U*_eq_(C).

## Supplementary Material

Crystal structure: contains datablock(s) I. DOI: 10.1107/S2056989025003317/vu2011sup1.cif

Structure factors: contains datablock(s) I. DOI: 10.1107/S2056989025003317/vu2011Isup2.hkl

Supporting information file. DOI: 10.1107/S2056989025003317/vu2011Isup3.cml

CCDC reference: 2443273

Additional supporting information:  crystallographic information; 3D view; checkCIF report

Additional supporting information:  crystallographic information; 3D view; checkCIF report

## Figures and Tables

**Table 1:** Hydrogen-bond geometry (Å, °) *Cg*1 and *Cg*2 are the centroids of the 4-bromo-2-chloro­phenyl (C1–C6) and phenyl (C10–C15) rings, respectively.

*D*—H⋯*A*	*D*—H	H⋯*A*	*D*⋯*A*	*D*—H⋯*A*
C6—H6⋯Cl1^i^	0.93	2.93	3.613 (2)	131
C9—H9⋯O2	0.93	2.50	2.848 (2)	102
C9—H9⋯O2^ii^	0.93	2.46	3.291 (2)	149
C15—H15⋯O2^ii^	0.93	2.58	3.371 (2)	143
C11—H11⋯*Cg*1^iii^	0.93	2.76	3.5716 (18)	147
C16—H16*B*⋯*Cg*2^iv^	0.97	2.79	3.6687 (17)	151
C18—H18*B*⋯*Cg*2^v^	0.97	2.85	3.7194 (17)	150

Symmetry codes: (i) 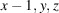; (ii) 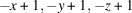; (iii) 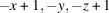; (iv) 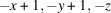; (v) 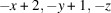.

**Table 2:** Experimental details

Crystal data	
Chemical formula	C_20_H_20_BrClO_3_
*M*_r_	423.72
Crystal system, space group	Triclinic, *P* 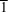
Temperature (K)	296
*a*, *b*, *c* (Å)	7.5850 (7), 9.825 (1), 12.8466 (13)
α, β, γ (°)	87.176 (3), 85.069 (3), 82.934 (3)
*V* (Å^3^)	945.85 (16)
*Z*	2
Radiation type	Mo *K*α
μ (mm^−1^)	2.33
Crystal size (mm)	0.32 × 0.27 × 0.24

Data collection	
Diffractometer	Bruker *SMART* APEXII CCD
Absorption correction	Multi-scan (*SADABS*; Krause *et al.*, 2015 ▸)
*T*_min_, *T*_max_	0.476, 0.570
No. of measured, independent and observed [*I* > 2σ(*I*)] reflections	14284, 4721, 4105
*R*_int_	0.035
(sin θ/λ)_max_ (Å^−1^)	0.668

Refinement	
*R*[*F*^2^ > 2σ(*F*^2^)], *wR*(*F*^2^), *S*	0.027, 0.066, 1.03
No. of reflections	4721
No. of parameters	227
H-atom treatment	H-atom parameters constrained
Δρ_max_, Δρ_min_ (e Å^−3^)	0.39, −0.45

Computer programs: *APEX2* and *SAINT* (Bruker, 2017 ▸), *SHELXT* (Sheldrick, 2015*a* ▸), *SHELXL* (Sheldrick, 2015*b* ▸) and *Mercury* (Macrae *et al.*, 2020 ▸).

## supplementary crystallographic information

### 4-Bromo-2-chlorophenyl (2E)-3-[4-(pentyloxy)phenyl]prop-2-enoate Crystal data

**Table:**

C_20_H_20_BrClO_3_	*F*(000) = 432
*M**_r_* = 423.72	Prism
Triclinic, *P*1	*D*_x_ = 1.488 Mg m^−^^3^
Hall symbol: -P 1	Melting point: 458 K
*a* = 7.5850 (7) Å	Mo *K*α radiation, λ = 0.71073 Å
*b* = 9.825 (1) Å	Cell parameters from 4105 reflections
*c* = 12.8466 (13) Å	θ = 2.5–29.0°
α = 87.176 (3)°	µ = 2.33 mm^−^^1^
β = 85.069 (3)°	*T* = 296 K
γ = 82.934 (3)°	Prism, colourless
*V* = 945.85 (16) Å^3^	0.32 × 0.27 × 0.24 mm
*Z* = 2	

### 4-Bromo-2-chlorophenyl (2E)-3-[4-(pentyloxy)phenyl]prop-2-enoate Data collection

**Table:**

Bruker SMART APEXII CCD diffractometer	4721 independent reflections
Radiation source: fine-focus sealed tube	4105 reflections with *I* > 2σ(*I*)
Graphite monochromator	*R*_int_ = 0.035
Detector resolution: 1.02 pixels mm^-1^	θ_max_ = 28.4°, θ_min_ = 2.7°
φ and Ω scans	*h* = −10→10
Absorption correction: multi-scan (SADABS; Krause *et al.*, 2015)	*k* = −13→13
*T*_min_ = 0.476, *T*_max_ = 0.570	*l* = −17→17
14284 measured reflections	

### 4-Bromo-2-chlorophenyl (2E)-3-[4-(pentyloxy)phenyl]prop-2-enoate Refinement

**Table:**

Refinement on *F*^2^	Primary atom site location: structure-invariant direct methods
Least-squares matrix: full	Secondary atom site location: difference Fourier map
*R*[*F*^2^ > 2σ(*F*^2^)] = 0.027	Hydrogen site location: inferred from neighbouring sites
*wR*(*F*^2^) = 0.066	H-atom parameters constrained
*S* = 1.03	*w* = 1/[σ^2^(*F*_o_^2^) + (0.023*P*)^2^ + 0.4253*P*] where *P* = (*F*_o_^2^ + 2*F*_c_^2^)/3
4721 reflections	(Δ/σ)_max_ = 0.004
227 parameters	Δρ_max_ = 0.39 e Å^−^^3^
0 restraints	Δρ_min_ = −0.45 e Å^−^^3^
0.123 constraints	

### 4-Bromo-2-chlorophenyl (2E)-3-[4-(pentyloxy)phenyl]prop-2-enoate Special details

**Table:**

Geometry. All esds (except the esd in the dihedral angle between two l.s. planes) are estimated using the full covariance matrix. The cell esds are taken into account individually in the estimation of esds in distances, angles and torsion angles; correlations between esds in cell parameters are only used when they are defined by crystal symmetry. An approximate (isotropic) treatment of cell esds is used for estimating esds involving l.s. planes.

### 4-Bromo-2-chlorophenyl (2E)-3-[4-(pentyloxy)phenyl]prop-2-enoate Fractional atomic coordinates and isotropic or equivalent isotropic displacement parameters (Å^2^)

**Table:**

	*x*	*y*	*z*	*U*_iso_*/*U*_eq_	
Br1	0.18439 (3)	0.12617 (2)	1.03966 (2)	0.02099 (6)	
Cl1	0.69315 (6)	0.12132 (7)	0.70527 (4)	0.03690 (13)	
O3	0.78618 (16)	0.38029 (12)	−0.06342 (9)	0.0169 (2)	
O1	0.38962 (16)	0.12409 (12)	0.57472 (9)	0.0169 (2)	
O2	0.3931 (2)	0.35324 (13)	0.57369 (10)	0.0260 (3)	
C4	0.2485 (2)	0.12778 (16)	0.89331 (13)	0.0159 (3)	
C10	0.5853 (2)	0.35279 (16)	0.24860 (12)	0.0134 (3)	
C12	0.6902 (2)	0.24985 (17)	0.08324 (13)	0.0154 (3)	
H12	0.716007	0.172269	0.043468	0.018*	
C8	0.4799 (2)	0.23374 (17)	0.41618 (13)	0.0151 (3)	
H8	0.492031	0.148155	0.386742	0.018*	
C7	0.4187 (2)	0.24945 (17)	0.52630 (13)	0.0155 (3)	
C11	0.6237 (2)	0.23732 (17)	0.18630 (13)	0.0155 (3)	
H11	0.604072	0.151208	0.214998	0.019*	
C13	0.7190 (2)	0.37875 (17)	0.03838 (12)	0.0140 (3)	
C9	0.5182 (2)	0.34530 (17)	0.35835 (13)	0.0147 (3)	
H9	0.499875	0.427875	0.392337	0.018*	
C1	0.3422 (2)	0.12854 (16)	0.68166 (13)	0.0147 (3)	
C5	0.1161 (2)	0.12990 (18)	0.82517 (14)	0.0202 (4)	
H5	−0.002839	0.131054	0.850218	0.024*	
C3	0.4258 (2)	0.12611 (17)	0.85862 (13)	0.0184 (3)	
H3	0.512420	0.124852	0.905778	0.022*	
C15	0.6154 (2)	0.48017 (17)	0.20237 (13)	0.0154 (3)	
H15	0.590935	0.557698	0.242285	0.018*	
C14	0.6808 (2)	0.49489 (17)	0.09858 (13)	0.0156 (3)	
H14	0.699023	0.581115	0.069501	0.019*	
C17	0.8891 (2)	0.49610 (18)	−0.22259 (13)	0.0180 (3)	
H17A	0.802673	0.458591	−0.260963	0.022*	
H17B	0.997676	0.432518	−0.225787	0.022*	
C18	0.9276 (2)	0.63480 (18)	−0.27262 (13)	0.0186 (3)	
H18A	0.819067	0.698353	−0.266778	0.022*	
H18B	1.014894	0.670824	−0.234011	0.022*	
C2	0.4721 (2)	0.12635 (18)	0.75163 (14)	0.0176 (3)	
C16	0.8173 (2)	0.51254 (17)	−0.11028 (13)	0.0159 (3)	
H16A	0.902213	0.552149	−0.072138	0.019*	
H16B	0.706771	0.573970	−0.107005	0.019*	
C6	0.1655 (2)	0.13025 (18)	0.71820 (14)	0.0200 (4)	
H6	0.078790	0.131658	0.671066	0.024*	
C19	0.9971 (3)	0.6278 (2)	−0.38731 (14)	0.0263 (4)	
H19A	1.108096	0.567089	−0.393177	0.032*	
H19B	0.911805	0.589191	−0.425798	0.032*	
C20	1.0284 (3)	0.7680 (3)	−0.43626 (17)	0.0380 (6)	
H20A	1.074526	0.758169	−0.507796	0.057*	
H20B	1.112534	0.806870	−0.398383	0.057*	
H20C	0.917802	0.827400	−0.433463	0.057*	

### 4-Bromo-2-chlorophenyl (2E)-3-[4-(pentyloxy)phenyl]prop-2-enoate Atomic displacement parameters (Å^2^)

**Table:**

	*U*^11^	*U*^22^	*U*^33^	*U*^12^	*U*^13^	*U*^23^
Br1	0.02982 (11)	0.01924 (9)	0.01305 (9)	−0.00280 (7)	0.00343 (6)	−0.00147 (6)
Cl1	0.0158 (2)	0.0705 (4)	0.0242 (3)	−0.0090 (2)	0.00258 (18)	0.0020 (2)
O3	0.0247 (7)	0.0142 (6)	0.0112 (6)	−0.0028 (5)	0.0034 (5)	−0.0005 (4)
O1	0.0242 (6)	0.0139 (6)	0.0123 (6)	−0.0038 (5)	0.0028 (5)	0.0001 (4)
O2	0.0471 (9)	0.0141 (6)	0.0165 (6)	−0.0068 (6)	0.0047 (6)	−0.0028 (5)
C4	0.0220 (9)	0.0108 (8)	0.0141 (8)	−0.0016 (6)	0.0028 (6)	−0.0003 (6)
C10	0.0129 (8)	0.0148 (8)	0.0125 (8)	−0.0018 (6)	−0.0020 (6)	0.0005 (6)
C12	0.0177 (8)	0.0135 (8)	0.0149 (8)	−0.0010 (6)	−0.0008 (6)	−0.0026 (6)
C8	0.0171 (8)	0.0142 (8)	0.0139 (8)	−0.0019 (6)	0.0011 (6)	−0.0027 (6)
C7	0.0173 (8)	0.0147 (8)	0.0142 (8)	−0.0033 (6)	−0.0002 (6)	0.0022 (6)
C11	0.0170 (8)	0.0128 (8)	0.0166 (8)	−0.0031 (6)	−0.0006 (6)	0.0012 (6)
C13	0.0145 (8)	0.0161 (8)	0.0111 (8)	−0.0018 (6)	−0.0006 (6)	0.0000 (6)
C9	0.0150 (8)	0.0158 (8)	0.0135 (8)	−0.0015 (6)	−0.0010 (6)	−0.0019 (6)
C1	0.0206 (8)	0.0103 (7)	0.0125 (8)	−0.0015 (6)	0.0012 (6)	0.0009 (6)
C5	0.0162 (8)	0.0247 (9)	0.0186 (9)	−0.0022 (7)	0.0024 (7)	0.0028 (7)
C3	0.0190 (9)	0.0205 (9)	0.0162 (8)	−0.0041 (7)	−0.0024 (7)	−0.0006 (6)
C15	0.0189 (8)	0.0128 (8)	0.0147 (8)	−0.0025 (6)	−0.0003 (6)	−0.0034 (6)
C14	0.0192 (8)	0.0126 (8)	0.0147 (8)	−0.0024 (6)	−0.0001 (6)	0.0010 (6)
C17	0.0189 (8)	0.0211 (9)	0.0131 (8)	−0.0006 (7)	0.0011 (6)	−0.0013 (6)
C18	0.0171 (8)	0.0236 (9)	0.0144 (8)	−0.0022 (7)	0.0002 (6)	0.0022 (6)
C2	0.0146 (8)	0.0197 (9)	0.0184 (9)	−0.0043 (6)	0.0019 (6)	−0.0003 (6)
C16	0.0184 (8)	0.0151 (8)	0.0138 (8)	−0.0025 (6)	0.0012 (6)	0.0006 (6)
C6	0.0180 (8)	0.0253 (9)	0.0167 (9)	−0.0033 (7)	−0.0021 (7)	0.0021 (7)
C19	0.0214 (9)	0.0406 (12)	0.0145 (9)	0.0012 (8)	0.0016 (7)	0.0044 (8)
C20	0.0250 (11)	0.0560 (15)	0.0298 (12)	−0.0034 (10)	0.0016 (9)	0.0219 (10)

### 4-Bromo-2-chlorophenyl (2E)-3-[4-(pentyloxy)phenyl]prop-2-enoate Geometric parameters (Å, º)

**Table:**

Br1—C4	1.9009 (16)	C5—C6	1.393 (2)
Cl1—C2	1.7263 (17)	C5—H5	0.9300
O3—C13	1.3623 (18)	C3—C2	1.389 (2)
O3—C16	1.4424 (19)	C3—H3	0.9300
O1—C7	1.3858 (19)	C15—C14	1.389 (2)
O1—C1	1.3914 (19)	C15—H15	0.9300
O2—C7	1.200 (2)	C14—H14	0.9300
C4—C3	1.378 (2)	C17—C16	1.507 (2)
C4—C5	1.385 (3)	C17—C18	1.528 (2)
C10—C15	1.395 (2)	C17—H17A	0.9700
C10—C11	1.407 (2)	C17—H17B	0.9700
C10—C9	1.459 (2)	C18—C19	1.523 (2)
C12—C11	1.381 (2)	C18—H18A	0.9700
C12—C13	1.399 (2)	C18—H18B	0.9700
C12—H12	0.9300	C16—H16A	0.9700
C8—C9	1.341 (2)	C16—H16B	0.9700
C8—C7	1.460 (2)	C6—H6	0.9300
C8—H8	0.9300	C19—C20	1.523 (3)
C11—H11	0.9300	C19—H19A	0.9700
C13—C14	1.397 (2)	C19—H19B	0.9700
C9—H9	0.9300	C20—H20A	0.9600
C1—C6	1.380 (2)	C20—H20B	0.9600
C1—C2	1.387 (2)	C20—H20C	0.9600
			
C13—O3—C16	116.35 (12)	C15—C14—C13	119.26 (15)
C7—O1—C1	114.71 (13)	C15—C14—H14	120.4
C3—C4—C5	122.21 (16)	C13—C14—H14	120.4
C3—C4—Br1	118.70 (13)	C16—C17—C18	110.16 (14)
C5—C4—Br1	119.09 (13)	C16—C17—H17A	109.6
C15—C10—C11	117.68 (15)	C18—C17—H17A	109.6
C15—C10—C9	118.90 (15)	C16—C17—H17B	109.6
C11—C10—C9	123.42 (14)	C18—C17—H17B	109.6
C11—C12—C13	120.30 (15)	H17A—C17—H17B	108.1
C11—C12—H12	119.8	C19—C18—C17	113.57 (15)
C13—C12—H12	119.8	C19—C18—H18A	108.9
C9—C8—C7	118.63 (15)	C17—C18—H18A	108.9
C9—C8—H8	120.7	C19—C18—H18B	108.9
C7—C8—H8	120.7	C17—C18—H18B	108.9
O2—C7—O1	121.15 (15)	H18A—C18—H18B	107.7
O2—C7—O1	121.15 (15)	C1—C2—C3	120.49 (15)
O2—C7—C8	127.83 (15)	C1—C2—Cl1	119.66 (13)
O2—C7—C8	127.83 (15)	C3—C2—Cl1	119.84 (14)
O1—C7—C8	111.02 (14)	O3—C16—C17	109.47 (13)
C12—C11—C10	121.05 (15)	O3—C16—H16A	109.8
C12—C11—H11	119.5	C17—C16—H16A	109.8
C10—C11—H11	119.5	O3—C16—H16B	109.8
O3—C13—C14	124.48 (14)	C17—C16—H16B	109.8
O3—C13—C12	115.88 (14)	H16A—C16—H16B	108.2
C14—C13—C12	119.64 (15)	C1—C6—C5	120.49 (17)
C8—C9—C10	127.86 (16)	C1—C6—H6	119.8
C8—C9—H9	116.1	C5—C6—H6	119.8
C10—C9—H9	116.1	C20—C19—C18	112.52 (18)
C6—C1—C2	119.99 (15)	C20—C19—H19A	109.1
C6—C1—O1	119.64 (15)	C18—C19—H19A	109.1
C2—C1—O1	120.32 (15)	C20—C19—H19B	109.1
C4—C5—C6	118.32 (16)	C18—C19—H19B	109.1
C4—C5—H5	120.8	H19A—C19—H19B	107.8
C6—C5—H5	120.8	C19—C20—H20A	109.5
C4—C3—C2	118.49 (16)	C19—C20—H20B	109.5
C4—C3—H3	120.8	H20A—C20—H20B	109.5
C2—C3—H3	120.8	C19—C20—H20C	109.5
C14—C15—C10	122.07 (15)	H20A—C20—H20C	109.5
C14—C15—H15	119.0	H20B—C20—H20C	109.5
C10—C15—H15	119.0		
			
C1—O1—C7—O2	−4.4 (2)	C5—C4—C3—C2	−0.1 (3)
C1—O1—C7—O2	−4.4 (2)	Br1—C4—C3—C2	179.57 (12)
C1—O1—C7—C8	175.98 (14)	C11—C10—C15—C14	0.1 (2)
C9—C8—C7—O2	1.9 (3)	C9—C10—C15—C14	179.33 (16)
C9—C8—C7—O2	1.9 (3)	C10—C15—C14—C13	−0.4 (3)
C9—C8—C7—O1	−178.50 (15)	O3—C13—C14—C15	−178.89 (15)
C13—C12—C11—C10	−0.7 (3)	C12—C13—C14—C15	0.2 (3)
C15—C10—C11—C12	0.5 (2)	C16—C17—C18—C19	−178.75 (15)
C9—C10—C11—C12	−178.75 (15)	C6—C1—C2—C3	−0.1 (3)
C16—O3—C13—C14	−0.5 (2)	O1—C1—C2—C3	−177.82 (15)
C16—O3—C13—C12	−179.66 (14)	C6—C1—C2—Cl1	178.92 (13)
C11—C12—C13—O3	179.49 (15)	O1—C1—C2—Cl1	1.2 (2)
C11—C12—C13—C14	0.3 (3)	C4—C3—C2—C1	0.1 (3)
C7—C8—C9—C10	178.25 (16)	C4—C3—C2—Cl1	−178.89 (13)
C15—C10—C9—C8	179.46 (17)	C13—O3—C16—C17	−179.32 (14)
C11—C10—C9—C8	−1.3 (3)	C18—C17—C16—O3	−178.46 (14)
C7—O1—C1—C6	100.14 (18)	O1—C1—C6—C5	177.77 (15)
C7—O1—C1—C2	−82.15 (19)	C17—C18—C19—C20	178.03 (16)
Br1—C4—C5—C6	−179.64 (13)		

### 4-Bromo-2-chlorophenyl (2E)-3-[4-(pentyloxy)phenyl]prop-2-enoate Hydrogen-bond geometry (Å, º)

*Cg*1 and *Cg*2 are the centroids of the 4-bromo-2-chlorophenyl (C1–C6) and phenyl (C10–C15) rings, respectively.

**Table:**

*D*—H···*A*	*D*—H	H···*A*	*D*···*A*	*D*—H···*A*
C6—H6···Cl1^i^	0.93	2.93	3.613 (2)	131
C9—H9···O2	0.93	2.50	2.848 (2)	102
C9—H9···O2^ii^	0.93	2.46	3.291 (2)	149
C15—H15···O2^ii^	0.93	2.58	3.371 (2)	143
C11—H11···*Cg*1^iii^	0.93	2.76	3.5716 (18)	147
C16—H16*B*···*Cg*2^iv^	0.97	2.79	3.6687 (17)	151
C18—H18*B*···*Cg*2^v^	0.97	2.85	3.7194 (17)	150

Symmetry codes: (i) *x*−1, *y*, *z*; (ii) −*x*+1, −*y*+1, −*z*+1; (iii) −*x*+1, −*y*, −*z*+1; (iv) −*x*+1, −*y*+1, −*z*; (v) −*x*+2, −*y*+1, −*z*.
